# Effectiveness of proprotein convertase subtilisin/kexin type 9 inhibitors in managing hypercholesterolemia post-statin-associated immune-mediated necrotizing myopathy: report of five cases and literature review

**DOI:** 10.1007/s00296-025-05860-0

**Published:** 2025-04-18

**Authors:** Melina Yerolatsite, Nanteznta Torounidou, Nafsika Gerolymatou, Aikaterini Panteli, Nikolaos Koletsos, Maria Karakosta, George Zarkavelis, Paraskevi V. Voulgari

**Affiliations:** 1 Department of Medical Oncology, School of Health Sciences, Faculty of Medicine, University of Ioannina, Ioannina, Greece; 2 Department of Rheumatology, School of Health Sciences, Faculty of Medicine, University of Ioannina, 45110 Ioannina, Greece; 3 Department of Internal Medicine, University Hospital of Ioannina, Ioannina, Greece

**Keywords:** Myositis, Hmg Coa statins, Pcsk9 protein, Case reports

## Abstract

Immune-mediated necrotizing myopathy (IMNM), a type of inflammatory myopathy, is associated with anti-SRP or anti-3-hydroxy-3-methylglutaryl coenzyme A reductase (anti-HMGCR) antibodies, with statin use potentially inducing statin-associated IMNM (SAIMNM) due to HMGCR targeting. Proprotein convertase subtilisin/kexin type 9 (PCSK9) inhibitors may offer a safer alternative for lipid-lowering in these patients. This study aims to describe the clinical characteristics of SAIMNM patients and evaluate the safety of PCSK9 inhibitors after myositis onset. We present the clinical characteristics of five SAIMNM patients and evaluate the safety of PCSK9 inhibitors in these cases. Additionally, we conducted a literature review using four different databases (Medline/PubMed, Scopus, Cochrane and DOAJ) to summarize the available data on IMNM. While numerous articles discussed statin-induced myositis, we selected only those studies that addressed the treatment of dyslipidemia after the management of IMNM. All five patients were women, with four having a history of statin use. One statin-naïve patient was positive for anti-SRP antibodies, while the others had anti-HMGCR antibodies. After a mean follow-up of 18.2 months, creatine phosphokinase (CPK) levels dropped from 1028.6 IU/L to 135 IU/L, and LDL cholesterol levels decreased from 206.2 mg/dL to 87.2 mg/dL. All patients were treated with steroids (with a gradual dosage reduction), and four of the five received second line immunosuppressive therapy, such as intravenous immunoglobulin, methotrexate, azathioprine, and mycophenolate mofetil. No disease recurrence occurred after starting PCSK9 inhibitors. A review of seven studies (15 patients) showed a mean CPK of 1531.9 IU/L. 40% received steroids and another immunosuppressant. Statin rechallenge caused relapse in two cases, but PCSK9 inhibitors were well tolerated, with only one patient needing additional immunosuppression. Additionally, ezetimibe and bempedoic acid were used successfully in some patients. Finally, lipid levels appeared to be lower after treatment with PCSK9 inhibitors. Administration of PCSK9 inhibitors appears to be an effective and safe option for the treatment of dyslipidaemia in patients with IMNM.

## Introduction

Statins, one of the most frequently prescribed drugs worldwide, are the cornerstone of atherosclerosis management and cardiovascular disease prevention. Their mechanism of action is based on the inhibition of 3-hydroxy-3-methylglutaryl coenzyme A reductase (HMGCR), resulting in lowered cholesterol levels [1, 2]. While generally well tolerated, up to 25% of patients develop musculoskeletal side effects [1]. These effects range from asymptomatic elevation of creatine phosphokinase (CPK) to muscle pain, muscle weakness, myositis, and rhabdomyolysis. Most patients recover spontaneously after discontinuing statin treatment.

There are many different risk factors responsible for these symptoms. Specifically, it seems that statin-induced myopathy is dose- and type-dependent. The risk of myopathy is lower with fluvastatin, pravastatin, and pitavastatin, as these statins are not metabolized by CYP3 A4, reducing the likelihood of drug interactions. In contrast, the risk of muscle injury is significantly higher with statins extensively metabolized by CYP3 A4, such as lovastatin, simvastatin, and atorvastatin. Additionally, the risk is influenced by the patient's age and gender. For this reason, various mechanisms have been proposed to explain these symptoms, including genetic predisposition and supplementary vitamin D. Among them, immunologically mediated mechanisms are a common cause of muscle symptoms in these patients, even after statin withdrawal. [3].

Furthermore, statins can also induce an immune-mediated necrotizing myositis (IMNM) with antibodies against HMGCR [1, 4–6]. This condition is usually more severe, with significant proximal muscle weakness and strikingly elevated CPK levels that persist even after the drug is stopped. As the clinical outcome of statin induced IMNM can be significantly improved with immunosuppressive therapy, it is important to recognize and treat it promptly [4–7].

IMNM is an autoimmune disease that can be diagnosed through the presence of specific antibodies, primarily anti- HMGCR and anti- signal recognition particle (SRP). Anti-SRP-positive IMNM is most commonly diagnosed in patients in their 40 s or 50 s, and its risk factors are less well understood compared to anti-HMGCR-positive IMNM. In white populations, no specific HLA haplotype has been linked to anti-SRP-positive IMNM, whereas the DRB1 08:03 and DRB1 14:03 alleles have been associated with the disease in Japanese and Korean populations, respectively [4, 8].

In contrast, the primary risk factor for anti-HMGCR myositis is statin use, as the target of these autoantibodies is similar to that of statins. Statin exposure in these patients ranges from 15 to 65%, depending on geographic location and ethnicity, with lower rates in Asia, moderate rates in Europe, and higher rates in the USA. Age is also a factor, as 90% of patients with anti-HMGCR-positive IMNM over the age of 50 have been exposed to statins. As with other autoimmune diseases, genetic factors may influence susceptibility to anti-HMGCR-positive IMNM. In adults, the MHC class II allele DRB1 11:01 is strongly associated with a predisposition to anti-HMGCR-positive IMNM, while in children, the risk is linked to the DRB1 07:01 allele [4, 9, 10].

Muscle histopathological analysis of anti-HMGCR-positive patients reveals muscle fiber degeneration, upregulation of MHC-I, and macrophage infiltration, suggesting an antibody-mediated toxicity pathway. These autoantibodies promote inflammation, oxidative stress, and muscle atrophy by reducing IL-4 and IL-13, thereby impairing myoblast fusion. In vitro and animal studies confirm their pathogenic role, as purified IgG from affected patients induces muscle deficiency and necrosis. Complement activation is a key mechanism; however, C5 inhibitors have proven ineffective. [11].

Since patients with statin-induced IMNM often have a high risk of cardiovascular disease, a dilemma arises regarding the management of their dyslipidemia. Proprotein convertase subtilisin/kexin type 9 (PCSK9) inhibitors are agents that can be used as an alternative when statins are contraindicated. Given that PCSK9 inhibitors reduce HMGCR levels, they are considered a safe option for patients with statin-induced IMNM [12–18].

We present a series of cases from our department in which patients with IMNM were treated with PCSK9 inhibitors for dyslipidemia after their symptoms improved, with no relapse of myositis. Additionally, a systematic review of related cases was conducted. Although there are many articles discussing the management of statin induced IMNM, few address the specific dyslipidemia treatments that were chosen [12–18].

## Case-presentations

We present five female patients with IMNM, with a mean age of 65.8 years. The presentation of these cases is based on the Case-Based Review Standards (CABARET) [19]. Four of these patients had been receiving statin therapy for dyslipidemia, while only one had not. The patient who did not receive statin therapy tested positive for anti-SRP antibodies, whereas all other patients had elevated levels of anti-HMGCR antibodies. This evidence supports a correlation between anti-HMGCR antibodies and statin use. Atorvastatin was prescribed to all the patients, with a mean treatment duration of 50.5 months.

The clinical presentation varied among patients, although CPK levels were consistently elevated. The mean CPK level before starting PCSK9 inhibitor therapy was 1028.6 ± 749.43 IU/L, while the most recent measurement after myositis treatment and the use of PCSK9 inhibitors was within normal limits (135 IU/L). In addition, a muscle strength examination was conducted, and pathological findings were present in only two cases. In these two cases (1 st and 5 th patients), muscle strength returned to normal after treatment with PCSK9 inhibitors. Moreover, during the diagnostic evaluation, a whole-body computed tomography (CT) scan was performed to exclude the possibility of undiagnosed cancer. None of the five patients showed any signs of cancer.

Following the onset of myositis, statins were discontinued, and treatment with steroids and immunosuppressants was initiated. Specifically, all patients received methylprednisolone, and in some cases, additional immunosuppressants were required. Methotrexate (MTX) was the preferred drug. Depending on the case, methylprednisolone doses, before the PCSK9 initiation, varied from 4 mg/day to 40 mg/day One patient also required intravenous immunoglobulin (IVIG) therapy. In the patient with anti-SRP myositis (4 th patient), rituximab was necessary to achieve remission. The interval between the onset of myositis and the initiation of PCSK9 inhibitors varied depending on the patient’s symptoms. In one case (1 st patient), PCSK9 inhibitors were started simultaneously with MTX, whereas in another patient, they were initiated three months after the start of MTX treatment. LDL levels were also a critical metric in these cases. It was important to assess whether PCSK9 inhibitors were not only a safe but also an effective option for these patients. LDL levels decreased by more than 50% following treatment with PCSK9 inhibitors. It seems that after the onset of IMNM, the initiation of PCSK9 inhibitors is a safe and effective choice for these patients. Table 1 summarizes the data of our department's cases and outlines the characteristics of each.

**Table 1:** Characterisrics of patients from our department

No.	Sex (Age)	Autoantibodies	Statin (type)	Statin exposure (mo)	Time interval between disease beginning and PCSK9i initiation (mo)	PCSK9i medication	Other lipid-lowering medications	Time on PCSK9i medication (mo)	Treatment before PCSK9i initiation	Treatment after PCSK9i initiation	CPKmax (UI/L)	CPK before PCSK9i	CPK after PCSK9i	LDL before PCSK9i	Muscle strength before PCSK9i	Muscle strength after PCSK9i
1.	F (76)	Anti-HMGCR	Atorvastatin	24	2	Evolocumab	No	17	Methylprednisolone 40 mg/d	Methylprednisolone 2 mg/d, MTX 15 mg/w (MTX and PCSK9i were initiated concurrently	4045	2403	209	55	Arms 5/5, quadriceps 4/5	Arms 5/5, legs 5/5
2.	F (69)	Anti-HMGCR	Atorvastatin	48	21	Evolocumab	Ezetimibe	9	Methylprednisolone 4 mg/d	Methylprednisolone 4 mg/d	3638	223	141	86	Arms 5/5, legs 5/5	Arms 5/5, legs 5/5
3.	F (71)	Anti-HMGCR	Atorvastatin	106	4	Evolocumab	No	20	Methylprednisolone 40 mg/d, IVIG	Methylprednisolone 6 mg/d, MTX 10 mg/w	3185	510	150	60	Arms 5/5, legs 5/5	Arms 5/5, legs 5/5
4.	F (61)	Anti-SRP	No	0	12	Alirocumab	Ezetimibe	26	Methylprednisolone 12 mg/d, Rituximab (past medications: MTX	Methylprednisolone 2 mg/d, Rituximab	1277	1008	104	128	Arms 5/5, legs 5/5	Arms 5/5, legs 5/5
5.	F (52)	Anti-HMGCR	Atorvastatin	24	3	Evolocumab	Ezetimibe	19	Methylprednisolone 32 mg/d	Methylprednisolone 2 mg/d, MTX 10 mg/w (MTX was initiated 3 mo after PCSK9i)	10,000	999	73	107	Arms 5/5, Quadriceps 4/5	Arms 5/5, legs 5/5

## Methods

### Search strategy

Our search strategy was deliberately broad to ensure comprehensiveness and include all potential studies reporting cases of patients with statin induced IMNM, as well as the safety of replacing statins with PCSK9 inhibitors for managing dyslipidemia. The algorithm we used included all commonly relevant terms related to necrotizing autoimmune myositis (NAM) and statins. Specifically for NAM, we also used the following terms: idiopathic inflammatory myopathy, myotoxicity, statin induced necrotizing autoimmune myopathy, SINAM, HMGCR-associated myositis, necrotizing autoimmune myositis, NAM and proximal muscle weakness. Furthermore, we applied our algorithm to four different databases (PubMed, Scopus, Cochrane (Database of Systematic Reviews and CENTRAL) and DOAJ) to ensure a comprehensive retrieval of all the literature relevant to our study.

### Study inclusion and exclusion criteria

We included all articles available up to the search date (March 27, 2025) that focused on case reports or reviews of SINAM and the safety of replacing statins with PCSK9 inhibitors. Conversely, we excluded articles involving non-human subjects and those published in languages other than English or French.

### Article selection and data extraction

**Fig. 1:**
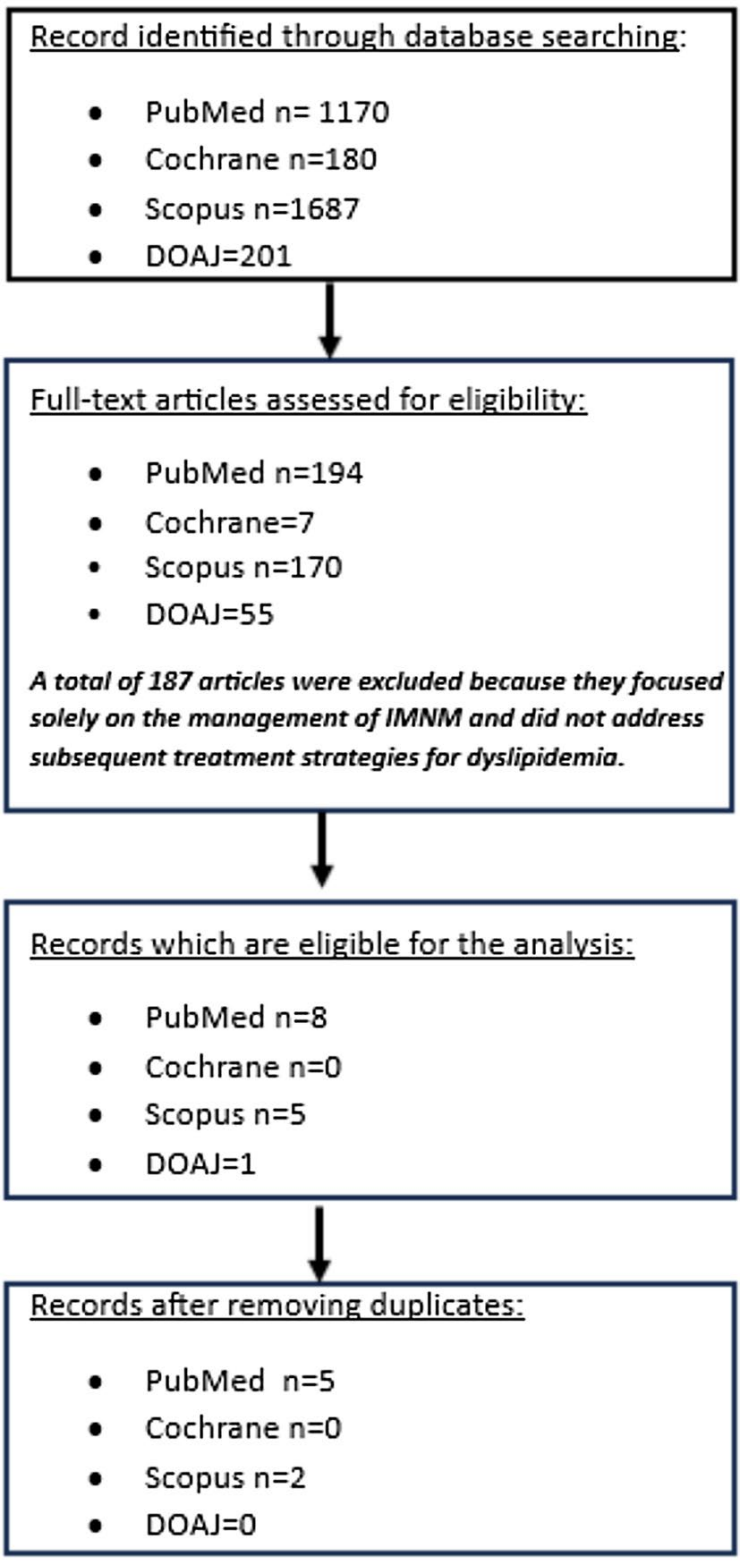
Flowchart depicting the search strategy followed in our analysis

Initially, we evaluated only the titles and abstracts, discarding articles that did not meet our criteria. We then thoroughly reviewed the remaining articles to assess their eligibility for our study (Fig. 1). The extracted data were first reviewed for accuracy before being entered into an electronic database. The database recorded various details, including the paper title, author, year of publication, patient gender, age, prescribed statin, duration of statin therapy, myositis treatment, treatment outcomes, medications used for dyslipidemia after myositis, whether there was a myositis flare, and whether LDL levels were lowered (Table 2).

**Table 2:** Characteristics of included studies

No	Author (Year)	Sex/Age	Type of statin	Past statin-associated medical history	Duration of statin treatment (yrs)	Initiation of symptoms	CPK max	Panel of antibodies for inflammatory disease	Anti-HMGCR	Myositis/Myopathy treatment	Treatment outcome	Lipid-lowering treatment after myositis	Myositis flare	LDL
1.	Villa [12]	F/65	Atorvastatin	No	4	During statin	5838 U/L	Neg	Positive (> 200)	Steroids, AZA	Improvement	Ezetimibe	No	Lower
2.	Malone [13]	/74	Atorvastatin	No	5	During statin	5700 U/L	Neg	Positive (200)	MTX, prednisone	Complete recovery	Evolocumab	No	NA
3**.**	Close [14]	F/68	Atorvastatin	Leg cramps	8	During statin	15,853 U/l	NA	Positive (> 200)	Steroids, IVIG, MTX- > MMF + steroids	Improvement	Statin rechallenge (Pravastin)	Yes	NA
4.	Tinakou [15]	F/50	NA	No	4	During statin	1141 IU/L	NA	Positive	MTX, steroids	Stable	Evocolumab	No	Lower
		F/55	NA	No	2	During statin	677 IU/L	NA	Positive	IVIG	Stable	Alirocumab	No	Lower
		F/43	NA	Myositis	15	During statin	677 IU/L	NA	Positive	MTX, steroids, IVIG	Stable	Evocolumab	No	Lower
		F/64	NA	No	2	During statin	981 IU/L	NA	Positive	AZA	Stable	Alirocumab	No	Lower
		F/65	NA	No	1	During statin	72 IU/L	NA	Positive	MTX, steroids	Stable	Alirocumab	No	Lower
		F/54	NA	No	13	During statin	3539 IU/L	Nain	Positive	MTX, IVIG	Stable	Evocolumab	No	Lower
		M/57	NA	No	3	During statin	121 IU/L	NA	Positive	Steroids, IVIG	Stable	Evocolumab	No	Lower
		M/56	NA	No	15	During statin	1205 IU/L	NA	Positive	Steroids	Stable	Evocolumab	Yes	Lower

No	Author (Year)	Sex/Age	Type of statin	Past statin-associated medical history	Duration of statin treatment (yrs)	Initiation of symptoms	CPK max	Panel of antibodies for inflammatory disease	Anti-HMGCR	Myositis/Myopathy treatment	Treatment outcome	Lipid-lowering treatment after myositis	Myositis flare	LDL
5.	Shuster S [16]	M/74	Atorvastatin	Myositis	13(intermittent efforts)	After cessation (2 yrs)	813 mg/dl	Neg	Positive (4000)	IVIG- > AZA + steroids	Improvement	Evolocumab	No	Lower
6.	Dios Garcias-Diaz [17]	M/57	Atorvastatin	No	2.2	During statin	249.2 μkat/L	Neg	Positive	Steroids, MTX,IVIG	Resolution	Evolocumab	No	Lower
		M/66	Atorvastatin	No	> 4.5	During statin	186.8 μkat/L	Neg	Positive	Steroids, AZA,IVIG	Resolution	Evolocumab	No	Lower
7.	Obreja E.[18]	F/67	Atorvastatin	No	6	During statin	720 mg/dl	Neg	–	steroid injection and iv	Free of symptoms	Statin rechallenge (pravastatin)	Yes with INM (started prednisone, MTX, IVIG)	Lower

## Results

Although many articles discuss statin associated necrotizing myositis, few address the appropriate lipid-lowering treatment that should be administered after the improvement of clinical symptoms. Through this literature review, only seven relevant case reports and one study summarizing details from ten patients with the same diagnosis were identified [12–18].

In the seven case reports, a total of fifteen patients with statin associated myositis were examined. The mean age of the patients was 61 years, and the average duration of statin use was 6.5 years. Atorvastatin was the most commonly administered statin. Myositis typically developed while patients were taking statins, although in some cases, it occurred after statin discontinuation [11–17]. In the majority of cases, myositis worsened after statins were stopped, supporting the autoimmune mechanism of the disease [13, 14, 16–18]. In Alvarez-Troncoso study, all patients received atorvastatin, with a mean dose of 50 mg and an average treatment duration of 20.8 months before symptom onset. Additionally, the mean time from symptom onset to drug discontinuation was 2.6 months [18].

The severity of symptoms varied among the patients. Some experienced mild symptoms, while others had more severe symptoms that endangered their lives, with some being unable to stand or walk. Specifically, symptoms included proximal muscle weakness and myalgias, and laboratory tests revealed elevated CPK levels, with the highest reported value being 5,838 IU/L. The mean CPK value was 1531.9 IU/lt. Elevated C-reactive protein (CRP) and myoglobinuria were also observed, and some patients were diagnosed with renal failure [12–18]. It is important to note that myositis in these cases expressed anti-HMGR antibodies, and none of the patients had other types of autoantibodies, except in Alvarez-Troncoso study, where 3 patients tested positive for antinuclear antibodies (ANA) [12–18]. This review also highlighted a diagnostic delay between the onset of symptoms and the detection of positive antibodies [18]. In all cases, electromyography was performed, revealing evidence of myopathy. For instance, in Shuster study, rare small myopathic units were observed in the gluteal muscles [16]. In some cases, muscle MRI was performed and necrotizing myopathy with inflammation was found [17, 18]. In all cases, a biopsy was performed, and the pathology report indicated interfascicular inflammation, muscle atrophy, and actively necrotic muscle fibers [12–18]. Specifically, in Close study, the biopsy additionally showed macrophage infiltration and extensive endomysial fibrosis [14]. In cases of anti-HMGR-myositis, a complete diagnostic evaluation must be performed. Other autoimmune diseases must be excluded, and therefore an autoantibody panel was conducted in each case report. Additionally, tests were performed to rule out diabetes and B12 deficiency. Hepatitis and HIV tests were also part of the diagnostic evaluation. Lastly, imaging tests were used to check for undiagnosed cancer [12–18]. In the review, three patients had cancer: two were diagnosed before developing myositis, and one developed cancer after the myositis diagnosis [18]. On the other hand, in the case reports, none of the patients were diagnosed with cancer [13–18].

All patients, both in the review and the case reports, were treated with steroids and a second immunosuppressant [12–18]. The exception was two patients in Tinakou E. study: one was treated only with IVIG, and the other with azathioprine (AZA) [14]. Forty percent of the patients were treated with steroids and only one other immunosuppressant. AZA was chosen for immunosuppression in 26.6% of patients. In the majority of patients, IVIG and MTX were selected as the immunosuppressants, specifically in 60% and 53.3% of the patients, respectively [11–17]. In one patient who was treated with steroids and three different immunosuppressants, mycophenolate mofetil (MMF) was one of the drugs administered at post-hospital discharge [14]. In the review, five patients received only MTX, three received MTX with IVIG, and one received only IVIG. At the one-year follow-up, all patients had achieved disease remission, with normal CPK levels and improvement in symptoms [18].

Available data concerning dyslipidemia treatment after the occurrence of statin associated myositis were presented in Table 2. Some case reports mention that a statin rechallenge was attempted, though the reappearance of myositis was observed [14, 16, 18]. Specifically, in Close study, a patient received a different type of statin for dyslipidemia. After a year of myositis, the patient was given a low dose of pravastatin instead of atorvastatin. However, a relapse of myopathy occurred, with symptoms of muscle weakness and elevated CPK levels, necessitating treatment with steroids, MTX and IVIG [14].

Similarly, in Obreja study, an attempt at rechallenge was made, but a myopathy flare was observed [18]. In Shuster study, several efforts were made to tolerate statin therapy using different types of statins, but each attempt led to the recurrence of myopathy-myositis symptoms. In the final attempt, myopathy symptoms reappeared, and the statin therapy was discontinued. Two years later, persistent proximal lower extremity muscle weakness was observed, leading to the diagnosis of necrotizing myositis [16].

In every other case report, a change in dyslipidemia treatment was made, with PCSK9 inhibitors considered the best option [12, 13, 15, 17]. In all cases, there was no relapse of myositis with PCSK9 inhibitors, except for one patient in Tinakou study, where active disease persisted, necessitating an escalation of immunosuppressant therapy [15]. Lipid levels appeared to be lower after treatment with PCSK9 inhibitors, indicating that this type of dyslipidemia treatment may be a safe choice for managing the cardiovascular risk in these patients [12, 13, 15, 17].

Finally, in the review, ezetimibe was prescribed to all patients. Additionally, two patients were prescribed PCSK9 inhibitors (evolocumab), and one patient received bempedoic acid. Both drugs were well tolerated, and the addition of PCSK9 inhibitors enhanced the lipid-lowering effect of ezetimibe [18].

## Discussion

Statins are one of the most common lipid-lowering treatments, and their mechanism of action involves the inhibition of HMGCR [1]. According to the European Society of Cardiology and American College of Cardiology/American Heart Association guidelines, statins are the first-line therapy for primary hypercholesterolemia and the prevention of atherosclerotic cardiovascular disease [20–22]. However, musculoskeletal side effects may occur during statin treatment, necessitating a postponement or cessation of therapy. The most prevalent adverse effects of statins occur in skeletal muscle and are typically referred to as statin associated muscle symptoms. These effects include myalgias (muscle aches without elevated CK levels), myopathy (muscle symptoms accompanied by CK levels exceeding 10 times the upper limit of normal), myositis (muscle inflammation), and rhabdomyolysis [1, 2, 23–25]. In some cases, these symptoms may worsen, leading to the onset of necrotizing myositis with positive anti-HMGCR antibodies [1, 2]. IMNM is a rare disease, with an estimated incidence of 2 to 3 cases per 100,000 patients treated with statins. It typically occurs in adults in their 50 s and 60 s who have been on statin therapy for 1 to 2 years, although it can also manifest in individuals with no prior history of statin use [5].

Takami’s study investigated the number of reported IMNM cases, identifying 172 instances of statin-associated IMNM from 145 patient reports between April 1, 2004, and March 31, 2023. Rosuvastatin was the most frequently implicated statin (34.3%), followed by pitavastatin (25.0%) and atorvastatin (22.1%). No cases were reported in patients using combination therapies containing statins. The number of reported cases increased from 3 in 2015 to a peak of 51 in 2019, then declined to 22 in 2020, 17 in 2021, and 21 in 2022. The estimated annual incidence rate remained consistently low across all statins, rarely exceeding 5 per 1,000,000 patients. [26].

IMNM consists of two subtypes: anti-HMGCR antibody IMNM, and anti-SRP antibody IMNM. Anti- HMGCR IMNM is often associated with statin exposure, while the cause of the latter type remains unclear [4–6]. This is further demonstrated by our cases, in which the patient who did not receive statins developed myositis with the presence of anti-SRP antibodies. In contrast, in the cases where statins were the cause, anti-HMGCR antibodies were expressed.

Statin induced IMNM is characterized by extremely elevated CK levels, often exceeding 10 times the upper limit of normal, typically around 45 times the upper limit of normal. Clinically, patients may experience myalgias along with bilateral, proximal, symmetric weakness, which can vary in severity. The detection of anti-HMGCR antibodies, demonstrates a sensitivity of 94.4% and a specificity of 99.3% for anti-HMGCR associated myopathy [5, 25, 27–29].

Muscle biopsy may show muscle fibers of varying sizes, along with signs of necrosis, regeneration, myophagocytosis, and abundance of macrophage infiltration. In up to 25% of cases, however, there may be an absence of muscle fiber necrosis or perimysial pathology [5, 30].

The pathogenesis of IMNM is not well understood. DRB1* 11:01 Class II HLA allele is a genetic risk factor for developing anti-HMGCR IMNM. A hypothesis for anti-HMGCR IMNM development suggests that genetic susceptibility and statin exposure which leads to increased HMGCR expression, might result in altered processing of the protein in muscle. Cryptic epitopes revealed by aberrant processing or statin binding, might be presented by DRB1* 11:01, leading to loss of tolerance to HMGCR and the development of anti- HMGCR antibodies. These antibodies recognize surface antigen on muscle cells activating the complement pathway and resulting in muscle cell necrosis. Regenerating muscle cells express the autoantigen and could perpetuate destruction of muscle cells even after the discontinuation of statins [31, 32]. Another theory is that statin inhibition leads to an accumulation of HMG-CoA reductase, which may directly cause muscle toxicity and cramping. These mechanisms likely interact rather than act independently, making the pathogenesis complex and not yet fully understood. [32, 33].

The review of the literature confirms that statin discontinuation is not enough to stop the progression of this myositis. A combination therapy with steroids and immunosuppressants is necessary [12–18]. Rituximab is used in the cases of refractory myositis [34]. Moreover, it seems that patients with anti-HMGCR myopathy show a strong response to immune suppression, highlighting the importance of broader antibody testing in individuals with myopathy [35, 36].

The main purpose of this literature review is to evaluate the treatment of dyslipidemia after IMNM. Most articles in the literature focus on the management of IMNM, but few address what happens afterward. This is crucial because the risk of cardiovascular events due to dyslipidemia remains significant even after the onset of IMNM. Therefore, it is essential to determine the appropriate next steps in treatment.

Rechallenging with the same or a different statin should be avoided, as it can trigger a disease flare. In Obreja Elena’s study, a case report described a mistaken diagnosis of statin-induced myalgia. A different statin was re-administered, leading to a recurrence of symptoms. The authors concluded that careful evaluation of patient symptoms is essential and that SAIMN should be considered in the differential diagnosis from the outset [37]. The literature review indicates that ezetimibe is the lipid-lowering medication prescribed in almost all cases. Its role involves lowering LDL-C by selectively inhibiting the Niemann-Pick C1-like 1 protein, which decreases intestinal cholesterol absorption. Additionally, ezetimibe inhibits macrophage migration, reduces vascular cell adhesion molecule 1 expression, and decreases levels of reactive oxygen species, potentially mitigating inflammation [38]. Although it can lower LDL-C levels by 15% to 20%, it appears to be inadequate as monotherapy for these patients [39].

PCSK9 inhibitors seem to be a safe and effective choice for these patients. PCSK9 is a proteolytic enzyme that indirectly regulates serum low-density lipoprotein cholesterol (LDL-C) levels by modulating the number of LDL receptors on cell surfaces. It plays a crucial role in hepatic LDL-C regulation. Although PCSK9 is primarily produced in the liver, it is also synthesized in extrahepatic tissues, including the kidney, intestine, and central nervous system, which may locally regulate LDL receptor expression. When PCSK9 binds to the LDL receptor before the LDL particle and receptor enter the hepatocyte, it prevents their separation within the endosome, causing the entire complex to be degraded in the lysosome. This results in premature degradation of the LDL receptor, reducing its presence on the cell surface and leading to elevated serum LDL-C levels as less LDL-C is cleared by the liver [40–42].

In Moriarty study, PCSK9 inhibitors have been proven to reduce LDL-C more effectively than ezetimibe [43]. The GAUSS-2 trial demonstrated evolocumab’s superior LDL-C reduction and lower myalgia rates compared to ezetimibe but lacked a blinded statin rechallenge. GAUSS-3 addressed this by incorporating a placebo-controlled rechallenge, confirming evolocumab’s greater efficacy at 24 weeks. Similarly, the ODYSSEY ALTERNATIVE trial found alirocumab more effective than ezetimibe in statin-intolerant patients, with fewer muscle-related events than atorvastatin [43–46]. Through our cases and the review of the literature, we demonstrated that this type of medication is a safe option, with none of the patients showing a relapse of myositis [12–18]. Only one patient required escalation of immunosuppressive therapy due to active disease [15]. LDL levels were also lower after treatment with PCSK9 inhibitors, and there were no reports of major cardiovascular events associated with the use of this category [12–18].

SAIMNM is a rare disease; therefore, ongoing clinician education and the expansion of medical knowledge about it are essential for early recognition and prompt intervention to reduce patient morbidity and improve outcomes.

This study provides important insights into anti-dyslipidemia treatment following statin-associated IMNM. While many studies in the bibliography focus on myositis management, few explore treatment options after myositis resolution. Specifically, there are many articles that examine the diagnostic procedure of SAIMNM [47–51, 53], the pathogenesis [50, 52, 58] and the therapeutic strategy for this disease [49, 51, 54, 57]. Additionally, some articles have been published due to the unusual presentation of SAIMNM [54–56]. However, due to its severity, only seven of them examine the anti-dyslipidemia treatment of these patients after recovering from myositis [12–18]. Given the high risk of complications due to dyslipidemia in these patients, it is essential to evaluate the next steps in their care. PCSK9 inhibitors have emerged as a safe and effective lipid-lowering therapy in this context. Moreover, understanding post-SAIMNM management remains crucial, as limited references address appropriate lipid-lowering treatments—making this the novel focus of our review.

## Conclusion

We presented the clinical characteristics of five SAIMNM patients treated with PCSK9 inhibitors and conducted a literature review to assess their efficacy and safety in this context. Statins are one of the main categories of medication that are prescribed daily, and for this reason, it is very important for clinicians to be aware of the possible side effects, especially those that may be life-threatening to patients. In cases of IMNMs, permanent discontinuation of statin therapy is mandatory, along with initiation of steroids and immunosuppressants. In these patients other types of lipid-lowering treatments must be administered. PCSK9 inhibitors appear to be not only safe but also an effective therapeutic choice.

## Data Availability

Data are available within the article.
